# Evaluation of Quality and Readability of Health Information Websites Identified through India's Major Search Engines

**DOI:** 10.1155/2016/4815285

**Published:** 2016-03-28

**Authors:** S. Raj, V. L. Sharma, A. J. Singh, S. Goel

**Affiliations:** ^1^Panjab University, Chandigarh 160014, India; ^2^Postgraduate Institute of Medical Education and Research (PGIMER), Chandigarh 160012, India

## Abstract

*Background*. The available health information on websites should be reliable and accurate in order to make informed decisions by community. This study was done to assess the quality and readability of health information websites on World Wide Web in India.* Methods*. This cross-sectional study was carried out in June 2014. The key words “Health” and “Information” were used on search engines “Google” and “Yahoo.” Out of 50 websites (25 from each search engines), after exclusion, 32 websites were evaluated. LIDA tool was used to assess the quality whereas the readability was assessed using Flesch Reading Ease Score (FRES), Flesch-Kincaid Grade Level (FKGL), and SMOG.* Results*. Forty percent of websites (*n* = 13) were sponsored by government. Health On the Net Code of Conduct (HONcode) certification was present on 50% (*n* = 16) of websites. The mean LIDA score (74.31) was average. Only 3 websites scored high on LIDA score. Only five had readability scores at recommended sixth-grade level.* Conclusion*. Most health information websites had average quality especially in terms of usability and reliability and were written at high readability levels. Efforts are needed to develop the health information websites which can help general population in informed decision making.

## 1. Introduction

Health promotion recognizes the vital importance of access of people to information. This is critical in achieving effective participation and empowerment of people and communities in health related activities [1]. Due to revolution in information and communication technology, the Internet has now become important source of health information both for consumers and for providers of health information [2–6]. Everything is now available on the click of a button. The developing countries like India have registered a year-on-year growth of 32% in number of Internet users. According to Internet and Mobile Association of India (IAMAI), India had more Internet users than United States by December 2014 [7]. According to Online Consumer Panel Report, 2011, 72% of Indian respondents used the Internet to access healthcare related information [8].

However, in order to make informed decisions regarding health, the available information should be reliable and accurate. On the Internet, due to freedom of information, almost anyone can create a website and offer expert advice regarding a host of topics. Research has shown that most contents on health information websites are not authored by medical professionals and not policed by any governing body or adhered to any ethical regulations [9]. Studies have shown that the quality of available health information on World Wide Web is not reliable [10–15]. Therefore, there is a risk that health information available may be misleading or dangerous [16]. Such information of dubious quality can do more harm than good [17]. Due to these rising concerns over the quality of health information available on the Internet, the Health On the Net foundation (HON Foundation) issued a code of conduct (HONcode) for health and medical information websites to address their reliability and usefulness. HONcode certification is an ethical standard aimed at offering quality, objectivity, and transparency of health information on websites by setting up a minimum set of principles. It is purely voluntary and demonstrates the intent of a website to publish transparent information [18].

Even if the contents of the websites are reliable, another important factor is the ability of users to understand those materials [19]. The available health information should be at the level which is easier to understand by the public and is not lost behind medical vocabulary. Readability of a written text is an objective measure of the reading skills an individual must possess to understand that material [20]. So, the present study was conducted to assess the quality and readability of health information websites on World Wide Web.

## 2. Methods

This cross-sectional study was carried out in month of June, 2014. The key words “Health” and “Information” were typed on two search engines namely “Google” and “Yahoo.” These search engines were taken as they are the top most search engine of the year 2014 [21]. In 2015, Yahoo Inc. formally entered a service agreement with search engine giant Google, Inc., where Google will provide Yahoo with search advertisements through Google's AdSense for Search (AFS) service, web algorithmic search services through Google's web search service, and image search services. By this agreement these two have collectively became the top search engine for accessing information [22]. Secondly, literature has shown that Google is the major search engine used to get health information [23].

There are few reports which documented that consumers often visit fewer than 25 topmost links found on a search, with most links being in the top five rank of the search results [24]. The searches were made through an India based IP address so as to get the most accessible sites in context to the study settings to make relevant contextual policy implications.

Therefore, we analyzed the top 25 web links per engine for their quality and readability. The criteria for inclusion of health information sites were that it should be written in English and offer general health information about various diseases. The repetitions, blogs, discussion groups, and specific journal article links were excluded. Out of 50 websites on health information (25 from each of the search engines), after exclusion, 32 websites were evaluated as follows. The presence of HONcode certification on the evaluated websites was also noted.

List of websites evaluated for quality and readability in the study (arranged in alphabetical order) is as follows:

 
http://www.aarogya.com/
 
http://www.cdc.gov/
 
http://www.clevelandclinic.org/
 
http://familydoctor.org/
 
http://hardinmd.lib.uiowa.edu/
 
http://www.health.gov/
 
http://www.health.nih.gov/
 
http://www.healthcareguide.com/
 
http://www.healthfinder.org/
 
http://www.healthline.com/
 
http://www.healthyindia.org/
 
http://www.heathlibrary.com/
 
http://www.hifa2015.org/
 
http://www.indmedica.com/
 
http://www.intelehealth.com/
 
http://www.mayoclinic.org/
 
http://www.medicinenet.com/
 
http://www.medindia.net/
 
http://www.medlineplus.com/
 
http://www.mohfw.nic.in/
 
http://www.motherchildtrust.org/
 
http://www.netwellness.org/
 
http://www.nhp.gov.in/
 
http://www.nlm.nih.gov/
 
http://www.nrhm.gov.in/
 
http://www.onlymyhealth.com/
 
http://www.patient.co.uk/health/
 
http://www.thehealthsite.com/
 
http://www.union-imdp.org/
 
http://www.webMD.com/
 
http://www.who.int/
 
http://www.360living.in/

### 2.1. Quality

The LIDA Instrument (The LIDA Instrument, Version 1.2, 2007, Minervation Ltd., Oxford, UK) was used to assess the quality of these websites. It is a validated method of evaluating healthcare websites based on three important areas: accessibility, usability, and reliability [25].

#### 2.1.1. Accessibility

It is based on ease of accessibility of the websites. The websites should meet legal accessibility requirements, without restrictions and outdated HTML code. The maximum possible score for accessibility was 54.

#### 2.1.2. Usability

It depends upon the understandability of the content in the websites. The aspects assessed included clarity of presentation, consistency of web page design, functionality including intuitive browsing and search facilities, and engageability. The maximum possible score was 12.

#### 2.1.3. Reliability

It is based on the accuracy of the information on the websites. It includes regular updates, clear declaration of conflict of interests, rigorous methodology for content production, and output. The maximum possible score was 30.

The maximum total score of a website on LIDA tool was 96. The accessibility score was computed by filling in the web address of the site on a customized web platform (http://www.minervation.com/mod product/LIDA). A nine-item questionnaire was applied to evaluate usability and reliability. The responses were graded from 0 to 3 (0: never; 1: sometimes; 2: mostly; 3: always). The scores lower than 50% were low, between 50 and 90% were medium, and equal to and above 90% were taken as high.

### 2.2. Readability

Three readability formulas were used to assess the readability of the websites.

The Flesch Reading Ease Score (FRES) is as follows [26]:

(1) $$\begin{array}{c} RE=206.835-\left(\;1.015\times ASL\;\right)-\left(\;84.6\times ASW\;\right), \end{array}$$

where RE is readability ease; ASL is average sentence length (i.e., the number of words divided by the number of sentences); and ASW is average number of syllables per word (i.e., the number of syllables divided by the number of words).

The output score ranges from 0 to 100. The scores between 90.0 and 100.0 are considered easily understandable by an average 5th grader; scores between 60.0 and 70.0 are considered easily understood by 8th and 9th graders; scores between 0.0 and 30.0 are considered easily understood by college graduates. The higher the number is, the easier the text is to read.

The Flesch-Kincaid Grade Level (FKGL) is as follows [27]:

(2) $$\begin{array}{c} FKRA=\left(\;0.39\times ASL\;\right)+\left(\;11.8\times ASW\;\right)-15.59, \end{array}$$

where FKRA is Flesch-Kincaid reading age; ASL is average sentence length (i.e., the number of words divided by the number of sentences); ASW is average number of syllables per word (i.e., the number of syllables divided by the number of words).

FKRA score of 5 indicates that the available health information can be comprehended by fifth grade school level and a score of 9.3 means that a ninth grader would be able to read the document.

Simple Measure of Gobbledygook (SMOG) is as follows [28]:

(3) SMOG  grade=3+Square  Root  of  Polysyllable  Count.

SMOG Readability Formula estimates the years of education a person needs to understand a piece of writing.

To calculate these readability scores, the online Readability Text Consensus Tool was used [29]. This tool analyzes the text and calculates the number of sentences, words, syllables, and characters in the sample. A sample of three, 50 to 100-words was copied from the beginning, the middle, and the end of the page of the website, respectively, and was pasted into an online readability calculator program to check the readability scores. Accuracy of the online method has been confirmed by the prior comparison of automated and manual calculation [30]. Data entry and analysis were done using IBM Statistical Package for Survey Solutions version 16 (SPSS-16). *p* value of less than 0.05 was considered significant.

## 3. Results

Out of total of 32 health information websites assessed, 40% (*n* = 13) were sponsored by government and 35% (*n* = 11) were sponsored by commercial agencies. Health On the Net Code of Conduct (HONcode) certification was present on 50% (*n* = 16) websites (Table 1).

### 3.1. Quality

**Figure 1:**
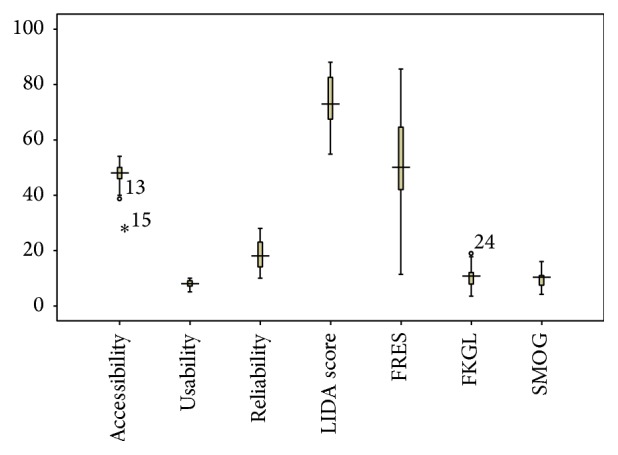
Box-and-whisker plot showing mean quality and readability scores of health information websites.

The mean LIDA score for accessibility was 47.2 (66.7%) out of possible 54. Eleven (34.4%) websites had high accessibility score (more than 90%). The average usability score was 8 (66.6%) out of total 12. None of the websites scored high on this parameter. The mean reliability score was 19 (63.3%) and only 5 (15.6%) websites scored more than 90% (high) on this parameter. The overall mean LIDA score for the websites was found to be 74.3 (77.4%) with SD 9.2 and interquartile range of 55–88, bordering on an overall medium score as shown in box-and-whisker plot in Figure 1 and Table 2. Only 3 (9.4%) websites scored high on LIDA score.

### 3.2. Readability

The mean FRES, FKGL, and SMOG score of health information websites were found to be 50.5, 10.2, and 9.6, respectively. Less than half (46.9%) of the websites had easy to read FRES score (60–100). Only 5 (15.6%) websites had FKGL, SMOG readability score at sixth-grade reading level as recommended to enhance the quality of educational materials for public [31] (Table 3).

Mean LIDA score was found to be significantly associated with the provider of the health information websites. Among the different readability scores, mean SMOG and FKGL score was also found to be significantly associated with the intended audience of these websites (Table 4).

## 4. Discussion

There is a wealth of health information and resources available on the Internet. However, the quality of health information available on the Internet is variable and dubious. The wrong information on these websites can put the health of the public “at risk” and can lead to serious consequences [32].

Search engines are the popular way to find health information on the Internet. According to Harris Poll, 2011, more than two-thirds (69%) of health information queries start with common search engine [33]. Therefore, we used main two search engines, namely, Google and Yahoo, to assess the quality and readability health information websites. Following the standard retrieval technique in e-health information searches, websites were explored beyond the first page of the links [24].

The present study found that quality of health information websites on parameters of accessibility, reliability, and readability by LIDA tool was quite different and average. Similar results have been found in other studies, wherein the available information on different medical conditions on the Internet has been found to be of low quality [10, 13, 15, 16, 34, 35]. Despite the ongoing revolution in the field of health information technology, efforts are needed to provide the general public with accurate and reliable health information which can help them in informed decision making regarding their health.

The methods to determine quality of medical content on websites are variable. Over the recent years, many tools are available to review health information websites. However, many of them are of doubtful utility [36]. We chose LIDA tool as it validated and covers all aspects of quality as accessibility, usability, and reliability and checks HTML and metadata for errors. The mean LIDA score (74.3) of health information websites in our study was found to be within the range of other studies [13, 15, 34]. Among the individual categories, the websites were easily accessible but usability and reliability score were low. So, further improvement is required especially in areas like transparency of sites, authorship by experts, update frequency, and citing of relevant sources. Government websites scored better in the LIDA tool assessment for health information.

Along with the quality, it is imperative that these websites should be written at a level which can be easily comprehended by the general public. It should be easily understood and accurate health information can enhance self-esteem, greater participation in care, and better informed decision making, thus leading to empowerment of the patients and their caregivers [37]. By using three readability formulas FRES, FKGL, and SMOG, we found that the majority of the health information websites were written in language which cannot be comprehended by a recommended sixth-grade reading level. The results were consistent with other studies [11, 14]. So, even if there is increased access to the Internet, the online health information is still hard to understand by majority of the people. Therefore, the administrators of the websites should be encouraged to improve the readability of the health information content so as to communicate a broad range of messages to a wide variety of audiences.

The present study found that only half of the websites were HONcode certified. Literature has shown that HONcode certification was present in 4%–30% of the websites [38, 39]. However, our study found no significant difference in quality of scores of websites irrespective of HONcode certification. Similar results have also been seen in other studies [15, 40]. This may be because of the fact that some of the websites may be suitable for certification but have not sought HONcode certification due to lack of awareness regarding this certification as this requires a voluntary application by the website managers.

The study has few important limitations. First, in the study we used only two search engines for retrieving information and hence cannot draw more general conclusions. However, the two search engines are the topmost engines used globally for searching about health information. Secondly, we used general term “health information” for evaluation of the websites instead of diseases specific key words since this paper was intended to assess the quality of information assessed by a disease-free (or apparently healthy) individual who would use the key words as used in the present study. The results might be different if we would have taken some diseases specific key words under the search strategy. Thirdly, the results are India specific as the “first 25” links will vary from country to country depending on the server used and hence will not be reflective of the health information websites browsed in another country. The country restriction was intentionally done so as to get the most accessible sites in context to the study settings to make context specific policy implications.

## 5. Conclusions and Recommendations

Most health information websites have average quality especially in terms of usability and reliability and are written at readability levels above the recommended sixth-grade reading level. Efforts are needed to develop the health information websites which can help general population in informed decision making.

## Figures and Tables

**Table 1:** Characteristics of health information websites (*N* = 32).

Characteristics	*n* (%)
Region	
Global	20 (62.5)
Indian	12 (37.5)
Intended audience	
Consumers	17 (53.1)
Healthcare providers	6 (18.8)
Both	9 (28.1)
Provider	
Government	13 (40.6)
NGO	8 (25)
Commercial	11 (34.4)
Health On the Net Code of Conduct (HONcode) logo	
Yes	16 (50%)
No	16 (50%)

**Table 2:** Box-and-whisker plot showing mean quality and readability scores of health information websites.

Domains (maximum score)	Accessibility (54)	Usability (12)	Reliability (30)	LIDA score (96)	FRES (100)	FKGL	SMOG
Mean (range)	47.2 (31–54)	8 (5–10)	19 (10–28)	74.3 (55–88)	50.5 (11.4–85.7)	10.2 (3.5–18.9)	9.6 (4.2–16)
SD	4.4	1.3	5.6	9.2	18.2	3.6	2.8

**Table 3:** Readability scores of health information websites.

Readability scores	*N* = 32, *n* (%)
FRES	
Easy (60–100)	10 (46.9)
Standard (50–59)	7 (21.9)
Difficult (0–49)	15 (31.2)
FKGL	
Up to grade 6	5 (15.6)
Grades 6–10	10 (31.2)
More than grade 10	17 (53.1)
SMOG	
Up to grade 6	5 (15.6)
Grades 6–10	10 (31.2)
More than grade 10	17 (53.1)

**Table 4:** Association between characteristics of websites with mean LIDA and mean readability scores.

Characteristics of websites	LIDA score mean ± SD	*p* value	FRES mean ± SD	*p* value	SMOG mean ± SD	*p* value	FKGL mean ± SD	*p* value
Provider								
Government	82.1 ± 5.2	0.00^*∗∗*^	55.6 ± 16.6	0.19	9.5 ± 3.1	0.83	9.8 ± 3.8	0.86
NGO	73.1 ± 6.7		53.1 ± 13.1		10.2 ± 2.2		10.7 ± 2.8	
Commercial	66 ± 6.7		42.6 ± 21.6		9.5 ± 3.1		10.3 ± 4.0	
Intended audience								
Consumers	71.3 ± 9.3	0.06	50.6 ± 18.7	0.22	9.5 ± 2.1	0.01^*∗*^	10.3 ± 3.6	0.01^*∗*^
Healthcare providers	81.3 ± 4.8		40.3 ± 8.7		12.2 ± 2		13.3 ± 2.6	
Both	75.3 ± 9.1		57.03 ± 20.1		8.2 ± 2.4		8.2 ± 2.8	
HONcode								
Yes	76.6 ± 8.6	0.14	57.2 ± 17.6	0.03^*∗*^	8.8 ± 2.4	0.09	9.3 ± 3.1	0.15
No	71.9 ± 9.4		43.7 ± 16.6		10.5 ± 2.9		11.1 ± 3.8	

^*∗*^ *p* significant at 0.01; ^*∗∗*^0.001 level.

## Additional Points

- This is the first study to assess both the readability and quality of general health information websites.
- Study indicates that websites are not meeting the recommended standards for readability and quality and thus raises concerns about their effectiveness in educating public.
